# The Use of hCG for Inducing Ovulation in Sheep Estrus Synchronization Impairs Ovulatory Follicle Growth and Fertility

**DOI:** 10.3390/ani11040984

**Published:** 2021-04-01

**Authors:** Macarena Bruno-Galarraga, Virginia Cano-Moreno, Beatriz Lago-Cruz, Teresa Encinas, Antonio Gonzalez-Bulnes, Paula Martinez-Ros

**Affiliations:** 1 Laboratorio de Reproducción de Rumiantes Menores, INTA Bariloche, San Carlos de Bariloche 8400, Argentina; brunogalarraga.m@inta.gob.ar; 2 Departamento Produccion y Sanidad Animal, Facultad de Veterinaria, Universidad Cardenal Herrera-CEU, CEU Universities, C/Tirant lo Blanc, 7, 46115 Alfara del Patriarca, Spain; mvirginia.cano@alumnos.uchceu.es; 3 Departamento de Toxicologia y Farmacologia, Facultad de Veterinaria, UCM, Ciudad Universitaria s/n, 28040 Madrid, Spain; beatrila@ucm.es (B.L.-C.); tencinas@vet.ucm.es (T.E.)

**Keywords:** eCG, estrus synchronization, hCG, ovulation, sheep

## Abstract

**Simple Summary:**

The present study, in view of the importance of finding an alternative hormone to eCG, aims to determine the response of sheep to hCG-based treatments. The findings indicate that the low fertility rates reported for protocols based on the administration of hCG for inducing ovulation during estrus synchronization in sheep may be related to a high occurrence of abnormal follicular growth patterns, disturbances, and retardments of ovulation and concomitant formation of follicular cysts in the treated females. These results preclude their practical application to induce ovulation concomitantly to estrous synchronization treatments.

**Abstract:**

Currently, there is an intense effort to find an alternative hormone to eCG to induce ovulation after estrus synchronization treatments in sheep. One of the proposed alternatives is based on the use of hCG, but the results are controversial since fertility rates are commonly affected. The present study aims to evaluate, therefore, the adequacy of hCG in protocols for the synchronization of estrus and ovulation. Ovarian follicle dynamics, occurrence of estrus behavior and subsequent ovulation, quality of corpora lutea, and pregnancy rate after controlled natural mating were assessed in two consecutive trials. The findings indicate that the low fertility rates reported for the protocols based on the administration of hCG for inducing ovulation during estrus synchronization in sheep may be related to a high occurrence of abnormal follicular growth patterns, disturbances, and retardments of ovulation and concomitant formation of follicular cysts in the treated females. These results preclude their practical application to induce ovulation concomitantly to estrous synchronization treatments.

## 1. Introduction

The management of reproduction in sheep mostly relies on protocols aiming at the induction and synchronization of estrus and ovulation for allowing out-of-season and/or synchronized lambing [1,2]. These protocols are based on the administration of either prostaglandin F_2α_ (for inducing the lysis of corpora lutea and subsequent estrus and ovulation) or progesterone or its analogs (for mimicking the activity of the corpus luteum) or a combination of both (for shortening the duration of the progestative treatment). Progesterone-based treatments usually include the administration of a single intramuscular dose of equine chorionic gonadotrophin (eCG) for mimicking the preovulatory luteinizing hormone (LH) surge and ovulation during the seasonal anoestrous to increase the percentage of twin pregnancies throughout the entire year and to adjust the interval between ovulation and semen deposition in fixed-time artificial insemination (TAI).

The future availability and trading of products containing eCG have been compromised by ethical concerns for animal welfare since the hormone is obtained from pregnant mares [2]. In consequence, there is intense research for alternative protocols, including hormones other than eCG. The substitutes are focused on other hormones, with the capacity for inducing the secretion of LH, like the gonadotrophin-releasing hormone (GnRH; [3,4,5]), or possessing LH activity in a similar way to eCG, like human chorionic gonadotrophin (hCG) or human menopausal gonadotrophin (hMG). The high similarity between the hormones hCG and LH causes their binding to the same LH receptor [6], and, in addition, the higher availability and lower cost of hCG have favored its use over hMG. Hence, hCG was earlier used for inducing ovulation during the anoestrus season in sheep [7] and tested afterward for its incorporation to protocols for estrus synchronization in heifers [8] and ewes [9]. These first data in sheep supported that hCG administration increased prolificacy but decreased fertility. More recent data support that sheep treated with hCG, in spite of inducing more synchronized ovulation than controls without gonadotrophin treatment, had a reduced pregnancy rate after TAI [10].

The present study, in view of the importance of finding an alternative hormone to eCG, aims to determine the response of sheep to hCG-based treatments. Ovarian follicle dynamics, occurrence of estrous behavior and subsequent ovulation, quality of corpora lutea, and pregnancy rate after natural mating were assessed in a first experiment. In view of the results obtained, a second trial was developed to establish more accurately the timing and characteristics of ovulation. These data may be useful for evaluating the adequacy of hCG in protocols for the synchronization of estrus and ovulation in natural breeding and also for ascertaining the causes for the reported reduced fertility after TAI.

## 2. Materials and Methods

### 2.1. Animals and Experimental Design

The study was approved by the Universidad CEU-Cardenal Herrera Committee of Ethics in Animal Research (report CEEA17/019) and involved 50 ewes, 3–5 years-old, with a mean body score of 3.0 ± 0.2, from the experimental farm of the University at Naquera (Valencia, Spain; latitude 39° N).

In the first trial, performed during the reproductive season (January), a total of 26 sheep were divided into two equal groups, which were treated with one intravaginal CIDR device (CIDR^®^ Ovis, Zoetis, Madrid, Spain) for five days plus one i.m. dose of 5 mg of prostaglandin F2α (Dinolytic^®^, Zoetis, Madrid, Spain) at CIDR withdrawal. Half of the sheep (*n* = 13; Group eCG) were treated with 400 IU of eCG (Foligon^®^, MSD Animal Health, Madrid, Spain) at CIDR removal whilst the remaining (*n* = 13; Group hCG) received 500 IU of hCG (Veterin-Corion^®^, Divasa Farmavic, Gurb-Vic, Spain) at 24 h after CIDR removal. The variables evaluated were occurrence of estrous behavior (determined with trained rams every 12 h from 24 to 84 h after CIDR withdrawal and allowing a single mating per ewe), ovarian follicle dynamics, and occurrence of ovulation or luteinization of follicles (determined by transrectal ultrasonography every 12 h from 24 to 84 h after CIDR withdrawal). Samples of jugular blood were collected concomitantly to ovarian ultrasonographies performed every 12 h with heparinized vacuum blood evacuation tubes (Vacutainer Systems Europe, Becton Dickinson, Meylan Cedex, France), centrifuged at 2000× *g* for 15 min and stored at −20 °C until assayed for estradiol-17β determination. Afterward, using ultrasonography, the presence of corpora lutea (CL), luteinized anovulatory follicles (FL), and follicular cysts (FC) was determined at Day 14 after CIDR removal, while a pregnancy diagnosis was performed at Day 35 after CIDR removal. Ultrasonographic scannings were performed with an Aloka SSD500 fitted to a 7.5 MHz linear-array probe (Aloka Co. Ltd., Tokyo, Japan). Estradiol-17β determination was performed after sample extraction using an enzyme immunoassay kit (Demeditec Diagnostics GmbH, Kiel-Wellsee, Germany); sensitivity was 1.4 pg/mL, and intra-assay variation coefficient was 5.7%. Plasma progesterone concentrations were measured using an enzyme immunoassay kit (Demeditec Diagnostics GmbH, Kiel-Wellsee, Germany). The sensitivity was 0.045 ng/mL, and the intra-assay variation coefficient was 5.4%.

In the second trial, which was also performed during the reproductive season (October), a total of 24 sheep were treated with one CIDR device and one dose of prostaglandin F2α at its withdrawal. Eight ewes were treated with 400 IU of eCG at CIDR removal (Group eCG), whilst 16 sheep received 500 IU of hCG at 24 h after CIDR removal (Group hCG). The timing of ovulation was determined by transrectal ultrasonography every 4 h, from 44 to 84 h, after CIDR withdrawal. The response to the treatments was also checked by evaluating the number of sheep showing estrous behavior at 24 h (i.e., at hCG treatment) and 48 h after CIDR removal. Afterward, using ultrasonography, the presence of corpora lutea (CL), luteinized anovulatory follicles (FL) and follicular cysts (FC), and progesterone levels were determined at Day 14 after CIDR removal.

### 2.2. Statistical Analysis

The effects of treatment (Groups eCG and hCG) on ovarian follicle dynamics, occurrence and onset of estrous behavior and ovulation, number of corpora lutea and other structures, secretion of estradiol and progesterone, and pregnancy rate were assessed by analyses of variance (ANOVA) and the chi-square test using SPSS 22.0 (IBM Corporation, New York, NY, USA). In brief, changes in the intergroup differences in numerical variables (number of follicles, plasma estradiol concentrations, timing of the different events and number of corpora lutea, and progesterone concentrations) were assessed for significance using a two-way analysis of variance (ANOVA), while their changes over time were assessed by a split-plot ANOVA for repeated measures, followed by Kruskal–Wallis tests when Levene’s tests showed nonhomogeneous variances. Intergroup differences in binomial variables (response to treatment) were assessed for significance using the chi-square test. Statistical analysis of results, expressed as percentages, was performed after arcsine transformation of the values for each individual percentage after normality testing of the data. All results in the main text and tables are expressed as mean ± SEM, and statistical significance was accepted from *p* < 0.05.

## 3. Results

In the first trial, all the 13 sheep in Group eCG showed estrous behavior and subsequent ovulation in response to the treatment (Table 1); twelve of them had at least one CL at Day 14 (92.3%), and 9 of them were pregnant at Day 35 (69.2%). On the other hand, only 8 of 13 ewes (61.5%) showed estrus signs in the group treated with hCG, but ovulation and subsequent presence of CL at Day 14 were observed in 11 of the 13 sheep (84.6%). However, only 5 of 13 (38.5%) were found to be pregnant. Similar results were found in the second trial, in which all the sheep in Group eCG but only 7 of 16 ewes in Group hCG (43.8%) showed estrous behavior prior to 48 h after CIDR removal. The ovarian scanning performed of the subsequent estrous cycle showed that all the ewes in Group eCG and 15 of 16 (93.7%) in Group hCG showed the presence of CL at Day 14 after CIDR removal.

**Figure 1:**
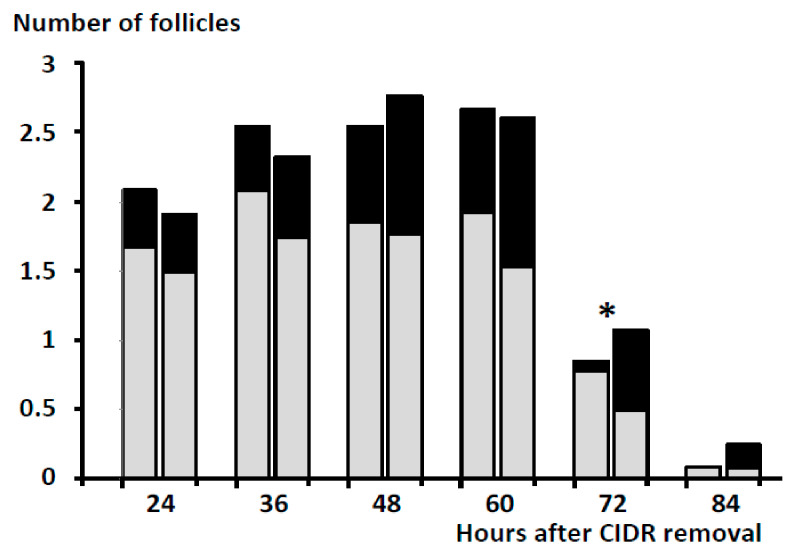
Mean number of follicles with a diameter of 3–4 mm (grey bars) or ≥5 mm (black bars) in sheep treated with Controlled Internal Drug Release (CIDR) devices for five days and 400 IU of eCG at CIDR removal + 5 mg of prostaglandin F2α (Group eCG; left bars at each time-point) or 500 IU of hCG at 24 h after CIDR removal (Group hCG; right bars at each time-point). Asterisk denotes significant difference (*p* < 0.05).

Scanning of follicle dynamics during the follicular phase in the first trial showed no significant differences between groups, excepting a significantly higher number of ovulatory-size follicles in Group hCG at 72 h after CIDR withdrawal (*p* < 0.05; Figure 1). All the sheep in Group eCG ovulated prior to 84 h after CIDR removal, while evidence of ovulation or luteinization at such moment was found in 11 of 13 sheep in Group hCG (84.6%).

**Figure 2:**
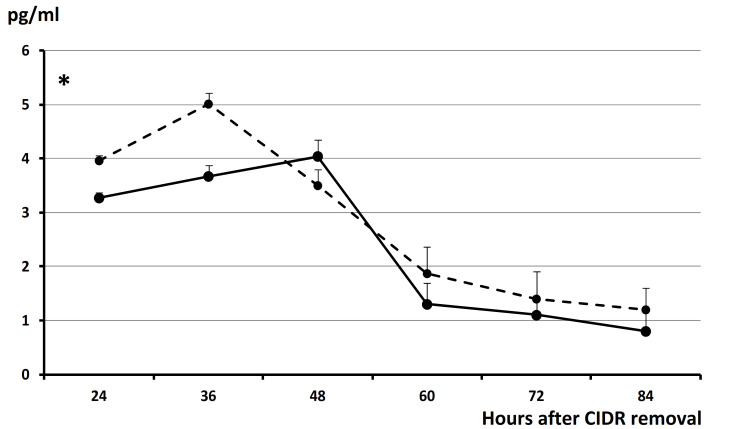
Mean (±SEM) plasma estradiol concentrations in sheep treated with Controlled Internal Drug Release (CIDR) devices for five days and 400 IU of eCG at CIDR removal +5 mg of prostaglandin F2α (Group eCG; discontinuous line) or 500 IU of hCG at 24 h after CIDR removal (Group hCG; continuous line). Asterisk denotes significant difference (*p* < 0.05). Data from ewes with high estradiol levels at 84 h were excluded (one ewe in Group eCG, with 11.4 pg/mL, and three ewes in Group hCG, with 27.9, 21.0, and 12.9 pg/mL).

Assessment of plasma estradiol concentrations during the follicular phase and periovulatory stages (24 to 84 h after CIDR removal) in Trial 1 showed similar patterns in both groups during the preovulatory stages but lower values around estrus in Group hCG (*p* < 0.05; Figure 2). Afterward, around 84 h, 12 of the 13 sheep in Group eCG (92.3%) had returned to basal values. At such moment, four sheep in Group hCG still maintained high plasma estradiol levels (30.8% vs. 7.7% in Group eCG; *p* < 0.05). At 84 h, one ewe in Group eCG and three ewes in Group hCG showed plasma estradiol higher than 10 pg/mL (11.4 pg/mL in Group eCG and 27.9, 21.0, and 12.9 pg/mL in Group hCG). All these ewes showed the presence of large nonovulatory follicles at ultrasound scanning.

The ovarian scanning performed at Day 14 after CIDR removal (Table 1) showed that in the first trial, a total of 11 of 13 sheep in Group hCG (84.6%) showed nonovulatory structures (FL+FC; mean of 1.8 ± 0.2), whilst a total of 4 of 13 sheep showed these structures in Group eCG (30.8%, mean of 1.0 ± 0.0; *p* < 0.05 for both parameters). In the second trial, nonovulatory structures were found in 2 of 8 ewes in Group eCG (25.0%; mean of 1.5 ± 0.1) and 10 of 16 ewes in Group hCG (62.5%; mean of 1.4 ± 0.2).

The detailed assessment of the onset of ovulation in the second trial showed that all the sheep in Group eCG ovulated between 56 and 72 h after CIDR removal (mean of 67.5 ± 0.4 h). Conversely, ovulation was found in only 7 out of 16 sheep in Group hCG (mean of 55.0 ± 0.3 h; *p* < 0.05) whilst the remaining 9 ewes evidenced no ovulation and early luteinization of some of the follicles at the last scanning at 84 h after CIDR removal.

## 4. Discussion

The results of the present study indicate that the efficiency of the administration of hCG, instead of eCG, for inducing ovulation during estrus synchronization in sheep is affected by the appearance of abnormal growth patterns in some of the presumptive ovulatory follicles. Such disruption causes deficiencies in the ovulatory process and the formation of follicular cysts, which may compromise fertility.

In this sense, the current study evidenced that all the ewes in both groups (eCG and hCG) displayed a similar number of gonadotrophin-responsive (≥3 mm) and preovulatory-sized (≥5 mm) follicles during the early stages of the follicular phase, induced by CIDR removal. In such a follicular phase, follicular development and periovulatory events in sheep treated with eCG were similar to previous works using the same protocol [3,11,12]. In brief, all sheep showed final follicular growth and onset of estrous behavior prior to 48 h, and almost all of them (95.2%, if joining both trials) ovulated around 70 h after CIDR removal and showed corpora lutea of good quality (as evaluated on Day 14 after CIDR removal). Afterward, the pregnancy rate with a single mating reached around 70%.

The group treated with hCG showed a higher appearance of ≥5 mm follicles after the injection of the hormone than the group treated with eCG, with significant differences at 72 h (at ovulatory timing). However, there was a low percentage of sheep displaying signs of estrous behavior and accepting mating in Group hCG (51.7%, if considering both trials). Evaluation of the ovaries at Day 14 after CIDR removal showed the presence of corpora lutea and adequate progesterone levels in almost all the treated sheep (around 90%, if considering both trials), but only around 40% of the sheep were pregnant. The presence of corpora lutea was concomitant with nonovulatory structures in around 70% of the sheep in Group hCG and around 14% in Group eCG. Assessment of periovulatory estradiol concentrations in the first trial and periovulatory follicular events in the second trial evidenced a deviation in ovulatory events, with the absence of ovulation and the presence of large nonovulatory follicles undergoing luteinization in around half of the ewes (56.2% in Group hCG; none in Group eCG) and the maintenance of high estradiol levels in around 30% of them (8% in Group eCG).

Hence, these results suggest a disruption of follicular development after hCG administration, which causes the growth and maintenance of large follicles that, in turn, may be involved in impairments of estrous behavior and ovulation and the lowering of the fertility rate. These findings support and would explain the decreased pregnancy rates previously reported after hCG treatments [9,10].

The absence of estrus and subsequent ovulation in some of the sheep treated with hCG (events determined by the rise in plasma estradiol concentrations caused by preovulatory follicles) suggest impairments of the quality of some of the large follicles developing after CIDR removal. Most of the plasma estradiol concentrations (around 90%) found in normal, nonstimulated, estrous cycles are synthesized by a large preovulatory follicle [13]. Plasma estradiol concentrations increase in the case of a high number of preovulatory follicles (e.g., in stimulated cycles [14]), so a lack of such increase would reflect a lower production of estradiol in all or some of the large follicles. The plasma estradiol concentration around the onset of estrous behavior can be considered an indicator of the population of healthy preovulatory follicles [14], since the production of estradiol reflects the quality of the preovulatory follicles [15]. In cows, it is well-known that healthy antral follicles contain high estradiol concentrations [16], while deficient follicles contain low estradiol concentrations [17] and are less able to release estradiol to blood flow [18]. In fact, a previous study in sheep showed that most (around 80%) of large anovulatory follicles are affected by functionality failures, either immaturity or early atresia, as indicated by a low intrafollicular estradiol concentration [19]. We can hypothesize a similar situation in the current study, precluding the appearance of estrous behavior and inducing a deviation in the ovulatory events. Possibly, the injection of hCG mimics a sharp discharge of LH, and such discharge induces luteinization or ovulation of the follicles. However, there are no previous changes in LH pulsatility and, therefore, in the expression of LH receptors of the granulosa cells of the preovulatory follicle [20], which are necessary for final follicle maturation, production of estradiol, and onset of estrous behavior [21].

Furthermore, lower follicle quality and an anomalous production of estradiol during preovulatory stages may also affect oocyte quality [22,23] and the expression of several hormones and signaling factors in oviduct and uterine secretions, which are crucial for early embryo development [24,25,26]. These factors also affect the fertility rate. These abnormalities may be caused by a deficiency in estradiol production during the preovulatory phase but also by excess during the early postovulatory phase, as was found in around 30% of the sheep treated with hCG in the present study. Such a hypothesis is supported by previous data assessing the estrogenicity of anovulatory follicles, which indicated that around 20% of them remained highly estrogenic and that their permanence beyond the occurrence of ovulation was related to lower fertility rates [19].

In conclusion, the low fertility rates reported for protocols based on the administration of hCG for inducing ovulation during estrus synchronization in sheep may be related to a high occurrence of abnormal follicular growth patterns, disturbances, and retardments of ovulation and formation of follicular cysts in the treated females. These results preclude their practical application to induce ovulation concomitantly to estrous synchronization treatments.

## Figures and Tables

**Table 1:** Percentage and timing of occurrence of estrus, characteristics of the ovarian response (in terms of corporal lutea (CL), plasma progesterone concentrations (P4), luteinized follicles (FL) and follicular cysts (FC), and pregnancy rate in sheep treated with Controlled Internal Drug Release (CIDR) devices for five days and 400 IU of eCG plus 5 mg of prostaglandin F2α at CIDR removal (Group eCG) or 500 IU of hCG at 24 h after CIDR removal (Group hCG).

Parameter	Group eCG First Trial (*n* = 13)	Group eCG Second Trial (*n* = 8)	Group hCG First Trial (*n* = 13)	Group hCG Second Trial (*n* = 16)
**Estrous behavior**				
Occurrence (%)	13/13 100% ^a^	8/8 100% ^a^	8/13 61.5% ^b^	7/16 43.8% ^b^
Timing (h)	46.1 ± 2.7		48.0 ± 4.7	
**Ovulation**				
Sheep bearing CL (%)	12/13 92.3%	8/8 100%	11/13 84.6%	15/16 93.7%
Mean number of CL	1.8 ± 0.3	2.1 ± 0.3	1.9 ± 0.2	2.2 ± 0.3
Mean P4 concentrations	2.3 ± 0.4	1.8 ± 0.3	2.4 ± 0.5	2.1 ±0.5
Sheep bearing FL (%)	3/13 23.1% ^a^	2/8 25% ^a^	8/13 61.5% ^b^	8/16 50% ^b^
Mean number of FL	1.0 ± 0.0 ^a^	1.5 ± 0.1	1.8 ± 0.2 ^b^	1.3 ± 0.1
Sheep bearing FC (%)	1/13 7.7% ^a^	0/8 0.0% ^a^	3/13 23.1% ^b^	2/16 12.5% ^b^
Mean number of FC	1.0	0.0 ^a^	1.7 ± 0.2	1.5 ± 0.1 ^b^
**Pregnancy**				
Occurrence (%)	9/13 69.2% ^a^		5/13 38.5% ^b^	

Different superscripts indicate significant differences among treatments in the same trial (a ≠ b: *p* < 0.05).

## Data Availability

Data is contained within the article.
